# The Experimental Transmission of Cancer

**Published:** 1908-02-22

**Authors:** 


The Hospital

A Journal of

The Medical Sciences and Hospital Administration.

New Series. No. 49, Vol. II. [No. 1121, Vol. XLIII.] SATURDAY, FEBRUARY 22, 1908.

### THE EXPERIMENTAL TRANSMISSION OF CANCER.

Ever since the introduction of experimental methods into the investigation of cancer, innumerable attempts have been made to transplant human cancer into animals. But, in this country, at any rate, these efforts have not met with success, and it is the general opinion of British pathologists that such a proceeding is, at present, beyond our powers. In the *Journal of Experimental Medicine* for January 1908, Dr. Guthrie McConnell, of St. Louis, gives an account of two attempts made by him to inoculate a human breast-cancer into rats, together with a summary of a great number of experiments of this nature, collected largely from the literature of Germany, where this line of research has received more attention than elsewhere. These experiments are divided into two groups, the "successful" and the "unsuccessful"; and it is somewhat surprising to find that a large proportion of them are classed by their authors under the former heading. But on a careful examination of the results, it is not easy to accept this verdict. The records of the "successful" cases are very incomplete, being often confined to a simple assertion that inoculation was followed by the growth of a cancer. In other cases more details are given, and we are told that cellular tumours developed either at the site of inoculation, or, after intravenous injection, in some more remote part of the body such as the lungs, liver or kidney. To balance these there are a number of "unsuccessful" experiments, including those of Herzog, who for several years made repeated attempts to transplant human cancer but never achieved his object; and one is led to the belief that the tumours obtained in the "successful" cases were merely granulomata formed by the cells of the animal inoculated.

The common experience is that, when a fragment of human cancer is introduced into the peritoneal cavity or subcutaneous tissues of an animal, it soon becomes surrounded by the wandering cells of its new host, and, after an interval of weeks or months is destroyed by the cytolysins of the animal and entirely disappears. It is true that there may be for some time either at the site of inoculation, or wherever the inoculated cells have lodged, a tumour, giving the word its simplest meaning of a swelling; but this tumour arises not from the growth of the cancer cells, but from an aggregation of the animal's phagocytes summoned to the spot by positive chemiotaxis. If such a focus be examined under the microscope it will be found to consist of a central core of cancer cells, surrounded by lymphocytes and hyaline connective-tissue cells. As time goes on the cancer cells lose their staining properties, and gradually disappear, but the surrounding lymphocytes mingled with a little fibrous tissue may persist for months as a hard nodule.

Dr. McConnell's experiments consisted in the implantation of small pieces of a scirrhous cancer of the human breast into the tissues of the abdominal wall of two rats, one of which was full grown, the other four weeks old. In the first rat a small nodule appeared at the seat of inoculation four days after the operation; this increased slightly in size during the next week, and then remained stationary until the rat was killed four months later. Microscopical examination of the nodule showed a central mass of degenerated material surrounded by a zone of cells, probably lymphocytes, and an outer capsule of fibrous tissue. A few remnants of the cancerous cells and stroma could be recognised in the degenerated core. The course of events in the second, and younger, rat was rather different. As in the first case, a small nodule appeared at the site of inoculation, but this had disappeared by the end of the first week, and the animal was alive and well four months later.

The failure of these and similar experiments is not surprising in view of the wide gulf that separates man from the rodents. The first evidence of the physiological antagonism existing between individuals of different species was discovered long ago, when it was found that transfusion of human blood into animals, and *vice versa*, was followed by destruction of the foreign corpuscles by hæmolysins. This antagonism is not, however, confined to the blood, and the general principle emerges that all living tissues resist the introduction of any foreign body by means of anti- substances directly hostile to those bodies. We must include amongst these foreign bodies, not only bacteria and other parasites, and inorganic materials, but also the organs and tissues of animals not of the same species; that is to say, a rabbit would oppose the introduction into its peritoneal cavity of a piece of human liver, just as it would oppose the introduction of a colony of streptococci; the resistance would differ in degree only.

Plainly, then, the successful transplantation of cancer from man to animals depends on the breaking down of the natural resistance shown by animals towards all human material, whether cancerous or healthy. The destruction of a natural immunity towards certain bacteria has often been achieved. Thus fowls, which are naturally immune to anthrax, will contract the disease if placed in cold water for some hours immediately after inoculation with the bacilli. This is a typical instance of "lowered resistance," and the varying susceptibility of man to microbial diseases is merely an expression of the same sequence of events.

To lower resistance towards bacteria is, therefore, relatively easy. A short exposure to an unfavourable and depressing environment will paralyse the anti- substances, and render an immune animal susceptible. But in the case of cancer, which develops very slowly, a greatly prolonged exposure would be required, and it is not easy to see how this can be brought about. Nevertheless, there seems reason to hope that research along these lines may eventually meet with some measure of success. We must, therefore, summon our patience to meet the continued disappointments which have hitherto dogged the way of cancer-investigation.

